# Unveiling the Ultimate Meme Recipe: Image Embeddings for Identifying Top Meme Templates from r/Memes

**DOI:** 10.3390/jimaging11050132

**Published:** 2025-04-23

**Authors:** Jan Sawicki

**Affiliations:** Faculty of Mathematics and Information Science, Warsaw University of Technology, ul. Koszykowa 75, 00-662 Warszawa, Poland; jan.sawicki@pw.edu.pl

**Keywords:** memes, Reddit, image embedding, natural language processing, clustering, image captioning

## Abstract

Meme analysis, particularly identifying top meme templates, is crucial for understanding digital culture, communication trends, and the spread of online humor, as memes serve as units of cultural transmission that shape public discourse. Tracking popular templates enables researchers to examine their role in social engagement, ideological framing, and viral dynamics within digital ecosystems. This study explored the viral nature of memes by analyzing a large dataset of over 1.5 million meme submissions from Reddit’s r/memes subreddit, spanning from January 2021 to July 2024. The focus was on uncovering the most popular meme templates by applying advanced image processing techniques. Apart from building an overall understanding of the memesphere, the main contribution was a selection of top meme templates providing a recipe for the best meme template for the meme creators (memesters). Using Vision Transformer (ViT) models, visual features of memes were analyzed without the influence of text, and memes were grouped into 1000 clusters that represented distinct templates. By combining image captioning and keyword extraction methods, key characteristics of the templates were identified, highlighting those with the most visual consistency. A deeper examination of the most popular memes revealed that factors like timing, cultural relevance, and references to current events played a significant role in their virality. Although user identity had limited influence on meme success, a closer look at contributors revealed an interesting pattern of a bot account and two prominent users. Ultimately, the study pinpointed the ten most popular meme templates, many of which were based on pop culture, offering insights into what makes a meme likely to go viral in today’s digital culture.

## 1. Introduction

Memes are culturally resonant images often paired with text, essential in digital communication [1]. The Cambridge Dictionary defines a meme as “an image, video, piece of text, etc., that is copied and spread rapidly by Internet users, often with slight variations” (https://dictionary.cambridge.org/dictionary/english/meme (accessed on 1 February 2025)). Scientific definitions of a meme range from “text over an image” [2] to “infectious ideas” that replicate through the internet [3], evolving in various formats such as videos, images, or phrases [4]. Despite their simplicity, the virality of memes is shaped by factors like template design, textual content, and timing. A prominent platform for meme sharing is Reddit.

Reddit is a major social news and discussion platform with around 1.2 billion monthly active users as of February 2024 (source: https://backlinko.com/reddit-users (accessed on 1 February 2025)). In Q3 2024, it reported 97.2 million daily active users, a 47% year-over-year increase (source: https://apnews.com/article/5a9e32159a947094ae801ab107c950da (accessed on 1 February 2025)). Organized into thousands of user-created subreddits, these forums cover diverse topics and begin with “r/” (e.g., r/science, r/memes, r/technology). One of the most popular subreddits, r/memes (https://www.reddit.com/best/communities/1/ (accessed on 1 February 2025)), was created on 5 July 2008, and is dedicated to meme culture. Similarly, to many previously studied subreddits [5], r/memes aggregates many memes, only a fraction of which receives the top user attention measured in users’ upvotes. This sparks a crucial question: “What makes a meme go viral?”

This study seeks to extract the most popular meme patterns by identifying the most widely used meme templates within Reddit’s r/memes subreddit. To achieve this, we focus primarily on analyzing the visual content of memes, allowing us to bypass the complexities introduced by textual overlays, which can vary significantly across posts. By concentrating on the visual elements, we aim to uncover the intrinsic qualities of the meme templates themselves, such as their composition, visual style, and structure, which contribute to their widespread appeal and viral potential.

To enhance our analysis, we employ advanced techniques such as Visual Transformers, which have proven effective in processing and understanding image-based data. These models enable us to extract rich, high-level features from meme images, allowing for a deeper understanding of the visual aspects that make certain templates more popular than others. Additionally, image embeddings are used to represent each meme as a compact and informative vector, capturing the essence of its visual content. By comparing these embeddings, we can perform clustering to group similar meme together, identifying distinct clusters (templates) of memes.

The combination of these methods—Visual Transformers, image embeddings, and clustering—allows us to extract the top meme templates (clusters based on embeddings) and to identify recurring patterns within top meme templates, all described in Section 5. This not only provides insight into what makes certain meme templates popular but also informs strategies for creating memes with a higher likelihood of going viral. By understanding these patterns, we can offer concrete suggestions for crafting memes that resonate with audiences and increase their chances of gaining widespread attention and engagement.

## 2. Related Work

Memes have been widely studied in the literature. In particular, r/memes has already appeared as a main focus of a notable study [6], which investigated the predictability of viral memes based on their content using a dataset of image-with-text memes collected from Reddit during the initial outbreak of COVID-19. The research framed the task as a binary classification problem, defining viral memes as those in the top 5% of posts by upvotes. The authors employed various machine learning models, with a random forest classifier achieving the best performance, showing an AUC of 0.6804, accuracy of 0.6638, precision of 0.0854, and recall of 0.5897. Despite the relatively low precision, the model demonstrated a 70% improvement over random guessing. Key features influencing meme virality included gray content, image size, saturation, and text length, while COVID-19-related content, although prevalent in the dataset, had limited impact on prediction performance.

The study also explored the predictive power of image and text features, finding that both contributed incrementally to meme success. For example, using convolutional neural networks (CNNs) with only image inputs achieved an AUC of 0.63, comparable to the random forest results. The authors highlighted that content-based analysis alone provided reasonable predictive efficiency, though the integration of social network and community features—previously shown to significantly improve prediction—could enhance accuracy further.

Limitations of the study included the short data collection period, which restricted the generalizability of the findings and excluded temporal analyses or the identification of phenomena such as “sleeping beauties” (memes that gain popularity long after being posted). The authors suggested future research avenues to investigate these dynamic aspects and to focus on COVID-19-inspired memes specifically. That work underscored the potential of combining content-based features with social network analysis for a comprehensive prediction of meme virality.

However, the work did not provide any deeper explanation of which memes “go viral” and why. In our contribution, instead of building a black-box machine learning model, we provide a full explanation of how a particular result was achieved. Moreover, we share particular results to highlight which groups of memes based on meme templates achieve much higher results.

Another study focused particularly on the meme templates, using them “as a lens for exploring cultural globalization” [7].

It explored cross-lingual Internet memes to analyze global and local expressive repertoires, highlighting their “glocal” nature—where global templates were adapted to local contexts. The analysis identified three key dimensions of cross-cultural meme usage. First, meme templates alternate between bottom-up and top-down expressions: they emerge as grassroots cultural creations but become standardized through popular meme generators. This top-down shift shapes expressive norms, such as the ironic portrayal of happiness, and reflects a muted form of Americanization, where global (often American) pop culture serves as a background for locally meaningful content. Notably, Chinese memes diverge from this trend, relying primarily on local cultural resources.

Second, the study found meme templates to be representationally conservative but emotionally disruptive. Across languages, dominant social groups—men and majority ethnicities—are prominently represented, while women and minorities are marginalized through both underrepresentation and stereotypical portrayals. This conservatism limits discourse around identity. Simultaneously, meme templates disrupt emotional norms by favoring negative emotions, particularly anger, which is framed as sincere and central to expression, while happiness is diminished through irony and mockery. This emotional pattern contrasts with the positivity norms typical of social media platforms.

Lastly, the study identified an “individualism–collectivism puzzle”: emotional expressions in memes did not align with cultural stereotypes. English and German memes, often associated with individualism, distance personal emotions through cynicism and focus on public or stereotypical subjects. In contrast, Chinese and Spanish memes, typically linked to collectivism, emphasize personal emotions, with Chinese memes highlighting sadness and Spanish memes expressing sincere happiness and anger. This contradiction suggests that digital expressive forms like memes may compensate for emotional expressions absent in other cultural contexts or challenge established cultural value frameworks. Additionally, external factors, such as censorship in China, may shape these patterns. The study underscored the value of analyzing digital artifacts to understand cultural expressions beyond traditional self-reported studies.

While that study provided extensive insights accounting for meme templates, it focused on a relatively limited sample size (n = 4000). The conclusions primarily addressed the impact of cultural factors on meme behavior, rather than identifying the specific elements that contribute to a meme’s virality. This limited the scope of the findings, as the study did not explore in depth the broader dynamics of viral content creation or the factors that drive widespread engagement.

Both aforementioned studies tackled the importance of the meme analysis. This contribution is a continuation of those research papers, combining the popularity aspect of the first one with the template recognition of the second one. Furthermore, it applies big data methods to provide large-scale tangible results for “memesters”. In this contribution, we aim to not only find or analyze the most popular memes but build a recipe for the most popular memes, by extracting their templates. For this, we need a sufficient and representative dataset of memes.

## 3. Dataset

The dataset comprised over 1,593,125 meme submissions from r/memes, spanning January 2021 to July 2024 (inclusive). During preprocessing, we removed the memes that sparked minimal interest among users while giving equal representation to each period (month). This was accomplished by filtering the top 1000 posts per period (month) by their score. For this analysis, only the image part of each post was used. Therefore, all posts with unavailable images (deleted by user, removed by administration, or simply unreachable due to network issues) were skipped. Eventually, 37,124 memes were taken into account.

**Figure 1:**
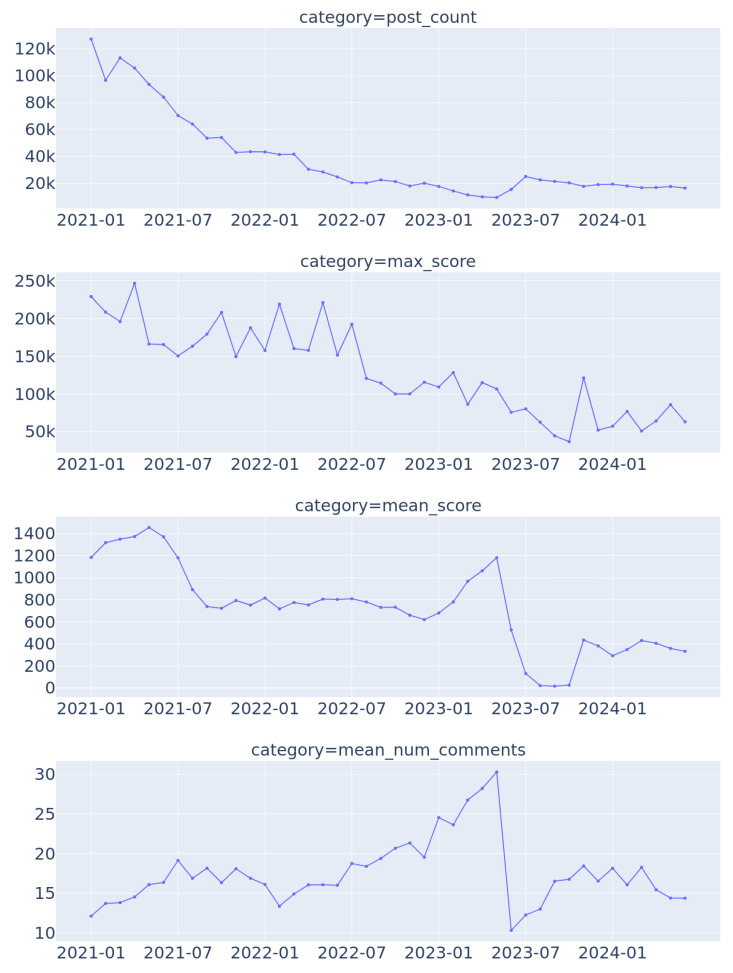
Post statistics in time (2021-mid 2024), top to bottom: post count, maximum score, mean score, mean number of comments.

Figure 1 illustrates the four basic statistics of posts in each month (period): post count, max score, mean score and mean number of comments. These statistics cover the overall behavior of posts frequency and popularity by users. “Score” refers to Reddit’s user appreciation mechanism [8]. First, the number of posts steadily declined from January 2021 until July 2022 and plateaued until July 2024. Second, the maximal score in each period seemed to start declining only after July 2022. These two observations are consistent with previous research [9] which indicated a “Reddit Blackout”.

The most fundamental changes appeared in the mean score and mean number of comments—a rapid downfall in May/July 2023. This major change overlapped with a major event in Reddit’s history.

The Reddit 2023 blackout (Wikipedia article (https://en.wikipedia.org/wiki/2023_Reddit_API_controversy (accessed on 1 February 2025)), Reddit thread 1 (https://www.reddit.com/r/OutOfTheLoop/comments/1aysjxs/what_is_up_with_the_aftermath_of_the_reddit/ (accessed on 1 February 2025)), Reddit thread 2 (https://www.reddit.com/r/OutOfTheLoop/comments/147fcdf/whats_going_on_with_subreddits_going_private_on/ (accessed on 1 February 2025)) represents a significant event in Reddit’s history, affecting both platform accessibility and data availability. In response to policy changes (source: https://www.reddit.com/r/OutOfTheLoop/comments/1aqxpfc/whats_going_on_with_the_current_state_of_the/ (accessed on 1 February 2025)), over 7000 subreddits temporarily switched to private mode (source: https://www.theverge.com/2023/6/12/23755974/reddit-subreddits-going-dark-private-protest-api-changes (accessed on 1 February 2025)), rendering their content inaccessible for data collection. This disruption had a direct impact on datasets used in this study, introducing gaps that could not be easily addressed or filled. Furthermore, the exact scope of the blackout remains unclear, as the number of affected subreddits and the nature of their content are not fully documented and evolved over time.

However, it is important to note that despite this large-scale event, none of the top memes analyzed in this study directly referenced the Reddit blackout. This may suggest that, while the blackout influenced data availability, it did not shape the most widely circulated memes. This may also suggest that the users did not upvote particular memes because they could not see them. Nonetheless, the limitations in data accessibility post-blackout introduced an inherent uncertainty in analyzing trends from mid-2023 onward.

Given that this challenge affects all research relying on Reddit data, it was not further addressed in this work. That said, the data analysis pipelines outlined in Section 4 and the findings presented in Section 5 extend beyond June 2023. From this point forward, results (especially statistics such as mean of posts score in Figure 1) should be interpreted with caution, as they reflect the structure of a post-blackout Reddit, where only a fraction of the platform’s previous content remained accessible for study.

## 4. Methods

Image analysis has historically relied on manual categorization or simple pixel-based comparisons, which fail to account for variations in resolution, cropping, and —especially popular in memes—overlaid text. Advances in machine learning, particularly in image embedding and clustering, have revolutionized this field. Vision Transformers (ViTs) [10,11] and pre-trained language models such as BLIP [12] and KeyBERT [13] now enable scalable, automated analysis of large visual datasets. However, the specific use of these tools to identify meme templates remains underexplored.

In this study, we focused on the application and analysis of the most advanced methods. The selection of these methods was informed by prior state-of-the-art reviews [14]. From an implementation perspective, we prioritized widely adopted models, as indicated by Hugging Face rankings based on the number of downloads and user feedback (https://huggingface.co/models?pipeline_tag=image-to-text&sort=downloads (accessed on 1 February 2025), https://huggingface.co/models?pipeline_tag=image-feature-extraction&sort=downloads (accessed on 1 February 2025)). Crucially, our choice of models was driven by the absence of a universally accepted standard for defining “best memes”. Given this ambiguity, our approach sought to generate high-quality meme outputs using the most robust and well-regarded methods currently available.

In this section, we present the methods in a pipeline which enables meme template enucleation with image embedding, clustering, and cluster analysis through NLP.

### 4.1. Image Embedding

To capture semantic similarity between meme images, we utilized the ViT-Base-Patch16-224-IN21k (https://huggingface.co/google/vit-base-patch16-224-in21k (accessed on 1 February 2025)) model, implemented via HuggingFace. The ViT model (Vision Transformer) [11,15] is a deep learning architecture designed for image processing tasks.

**Figure 2:**
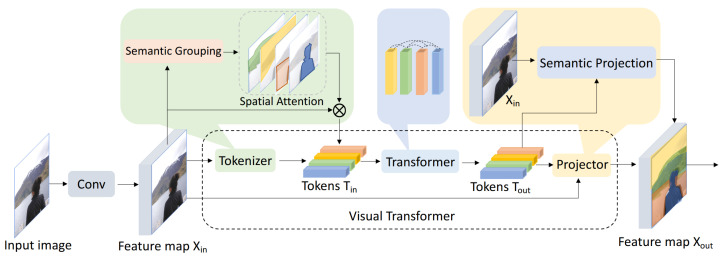
The original architecture of ViT [11]. The Visual Transformer (ViT) splits an input image into fixed-size patches, flattens them, and projects them into embeddings, adding positional information. These embeddings form a sequence processed by a standard Transformer encoder with multi-head self-attention and feed-forward layers. A special classification token is used to aggregate information and produce the final output for tasks like image classification.

The original architecture of ViT is shown in the model diagram in Figure 2. The diagram of a Vision Transformer (ViT) illustrates the following process: For a given image, the authors begin by using convolutional layers to extract low-level features. The resulting feature map is then passed into the Vision Transformer. First, a tokenizer groups pixels into a limited set of visual tokens, with each token representing a semantic concept within the image. Next, Transformers are applied to model the relationships between these tokens. Finally, the visual tokens are either directly used for image classification or projected back into the feature map for semantic segmentation.

Here, we specifically used the vit-base-patch16-224-in21k configuration. It operates by converting an image into a sequence of fixed-size non-overlapping patches (16 × 16 pixels in case of vit-base-patch16-224-in21k). These patches are linearly embedded into a vector space and then augmented with positional encodings, which help retain the spatial information of the image. This sequence of embedded patches is then passed through multiple transformer layers, where self-attention mechanisms allow the model to capture both local and global contextual relationships within the image. Unlike traditional convolutional neural networks, which focus on localized feature extraction through convolutional filters, Vision Transformers leverage self-attention to learn dependencies across the entire image, enabling them to understand intricate details and broader structures at once.

In the vit-base-patch16-224-in21k model, we have the following:

1. The “base” refers to the size of the transformer model, which is generally effective for medium-scale datasets.
2. “Patch16” indicates that the input image is divided into 16 × 16 pixel patches.
3. The number “224” refers to the image resolution (224 × 224 pixels)
4. The “in21k” indicates the model was pre-trained on a dataset of 21,000 classes (ImageNet21k), which significantly boosts its ability to generalize across various image domains.

The model has demonstrated its capacity to encode a variety of visual cues and fine-grained details, a key factor in its use for meme analysis in previous studies [16,17]. The ability of ViTs to understand global context helps them outperform traditional CNNs in certain image-based tasks that require the synthesis of multiple features and their interrelationships.

The choice of this particular version of the model, i.e., google/vit-base-patch16-224-in21k, was motivated by (1) its popularity in science and recent application related to memes [16], (2) its open-source availability on HuggingFace, (3) the size of the model, i.e., the “in21k” version of the model was trained on 14 million instead of 1 million images, which gives a wider context of understanding (potentially including meme caveats), (4) its versatility for image sizes and built-in rescaler being able to handle images of different sizes, and (5) the “base” version was chosen to speed up the computation given the large dataset.

### 4.2. Clustering

Since the end goal was to find meme template, a grouping of all memes was required. Such grouping is most commonly performed with clustering methods, among which K-means is the most popular one [5,18,19].

K-means clustering of image embeddings is an unsupervised learning technique used to group similar images based on their feature representations. After extracting high-dimensional embeddings from images—often using models like Vision Transformers (ViT) or convolutional neural networks—K-means partitions these embeddings into K clusters by minimizing the variance within each cluster. The algorithm iteratively assigns each embedding to the nearest cluster centroid and updates the centroids based on the mean of the assigned points. This method effectively groups images with similar visual patterns or semantic content, making it useful for tasks like image organization, retrieval, or discovering underlying structures in large, unlabeled image datasets. K-means has previously been applied to Reddit text data [5,20]. Here, we explored its usage on image embeddings.

Clustering was performed on the image embeddings using the K-Means algorithm. Several experiments with different numbers of clusters were considered (i.e., *K* = 500, 1000, 2000, 5000, and 10,000). Each of the setups was evaluated. The number 1000 balanced the granularity and in-cluster similarity producing the most consistent clusters by meme template used. This number is further addressed in Section 5.5.

### 4.3. Cluster Description

The next step of the pipeline was purely a “quality-of-life” improvement. The clusters already represented the meme templates. For easy interpretation and results comprehension, the clusters were further described with image captioning.

BLIP (Bootstrapping Language-Image Pre-training) [12] is a multimodal framework designed for image captioning, which combines visual and textual information to generate contextually rich and accurate descriptions of images. Central to the BLIP model is its vision encoder, typically implemented as a Vision Transformer (ViT), which extracts detailed features from the input image. These features are subsequently processed by a language model to generate captions that align closely with human-like interpretations of the image content.

The key strength of BLIP lies in its pre-training strategy, which integrates two components: vision–language contrastive learning and image–text matching. The contrastive learning objective optimizes the alignment between visual and textual representations, while the image–text matching task enhances the model’s ability to distinguish between correct and incorrect image–text pairs. These strategies work synergistically to improve the model’s cross-modal understanding, resulting in the generation of captions that are not only accurate but also context-aware, capturing both the objects and their interrelationships, actions, and implicit semantics within the image.

BLIP’s capacity to simultaneously process both visual and textual information enables it to generate descriptions that align with human interpretation, excelling in complex tasks such as visual storytelling, scene interpretation, and deep scene understanding. These tasks require a nuanced grasp of both the image content and the contextual information surrounding it, and BLIP’s multimodal framework is well suited to handle these challenges.

**Figure 3:**
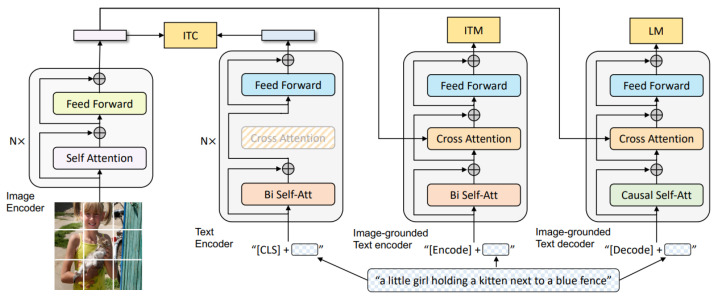
A high-level diagram of BLIP from the original paper [12]. The BLIP model uses a Vision Transformer (ViT) as its image encoder, where an image is split into patches, linearly embedded, and processed through multiple self-attention layers to capture global visual relationships. The ViT outputs a sequence of visual tokens, including a special [CLS] token summarizing the image representation. This representation is then aligned and integrated with text embeddings through a multimodal transformer for unified vision–language tasks.

The architecture of BLIP (see high-level diagram in Figure 3) employs a multimodal encoder–decoder framework, which operates in one of three configurations during its pre-training process. In the first configuration, the unimodal encoder is trained using an image–text contrastive (ITC) loss, aligning visual and textual representations. In the second configuration, the image-grounded text encoder incorporates additional cross-attention layers to model interactions between vision and language, and is trained with an image–text matching loss to distinguish positive from negative image–text pairs. In the third configuration, the image-grounded text decoder replaces the bi-directional self-attention layers with causal self-attention layers while maintaining shared cross-attention layers and feed-forward networks with the encoder. The decoder is trained with a language modeling loss, enabling it to generate coherent captions based on the visual input.

Through this sophisticated pre-training process, BLIP achieves exceptional performance in generating detailed, coherent, and contextually relevant image captions, which makes it a powerful tool for tasks requiring in-depth image interpretation and analysis.

In meme template captioning, BLIP’s capability to generate rich and context-aware descriptions enhances the intuitive interpretation and analysis of such visual content. Therefore, here BLIP was used to generate concise meme descriptions that could be further processed.

To further simplify and regularize the descriptions, keyword extraction was applied. This allowed the most salient and relevant terms to be highlighted, offering a concise representation of the image’s core themes. For this purpose, the captions were processed with KeyBERT.

KeyBERT is a state-of-the-art keyword extraction model that leverages BERT (Bidirectional Encoder Representations from Transformers) embeddings to identify the most salient and relevant terms within a given text. By utilizing BERT’s deep contextual understanding, KeyBERT can effectively extract keywords that encapsulate the core themes of the text, offering a concise representation of its central ideas. This process is particularly beneficial in scenarios where longer, complex descriptions need to be simplified and regularized. In the context of meme analysis, for example, KeyBERT distills the full descriptions of meme templates into a set of focused keywords that succinctly capture their underlying themes. These extracted keywords serve as an efficient shorthand for understanding the content, enabling quicker and more accessible analysis. Through this approach, KeyBERT plays a crucial role in streamlining the process of summarizing and interpreting textual information, making it more digestible for subsequent analysis and discussion.

KeyBERT’s role in this pipeline was to distill the longer, more complex descriptions from BLIP into a set of focused keywords. These keywords acted as an efficient shorthand for understanding the central ideas of each meme template, streamlining further analysis and discussion.

### 4.4. Template Identification

Manually browsing the results showed that many clusters were impure and contained memes from different templates. This sparked the need for homogeneity ranking and extracting the purest clusters to further analyze. Clusters were evaluated for homogeneity using cosine similarity measures.

Cosine similarity is a metric used to measure the similarity between two image embeddings by calculating the cosine of the angle between their corresponding vectors in a high-dimensional space. It focuses on the orientation rather than the magnitude of the vectors, making it effective for comparing image features regardless of scale differences. This is particularly useful in tasks like image retrieval or clustering, where identifying similar visual content is crucial. The formula for cosine similarity between two vectors *A* and *B* is given by:

$$cosine\_similarity=\frac{A\cdot B}{\left\|A\|\|B\right\|}$$

where A·B represents the dot product of the vectors, and ‖A‖ and ‖B‖ denote their Euclidean norms.

Although there are other metrics to evaluate clustering (e.g., silhouette score) the choice of cosine similarity was motivated by previous studies. although cosine similarity has been criticized [21], it is still widely used for image embedding comparison [22,23,24] as well as a multimodel application on memes with text and image [25].

The most homogeneous clusters, representing distinct used templates could finally be analyzed to extract the “top” meme templates on Reddit.

## 5. Results and Discussion

Having presented the method, we move to the results. Before presenting the most homogeneous clusters, i.e., the best meme templates, we present preliminary observations.

### 5.1. Authors

**Figure 4:**
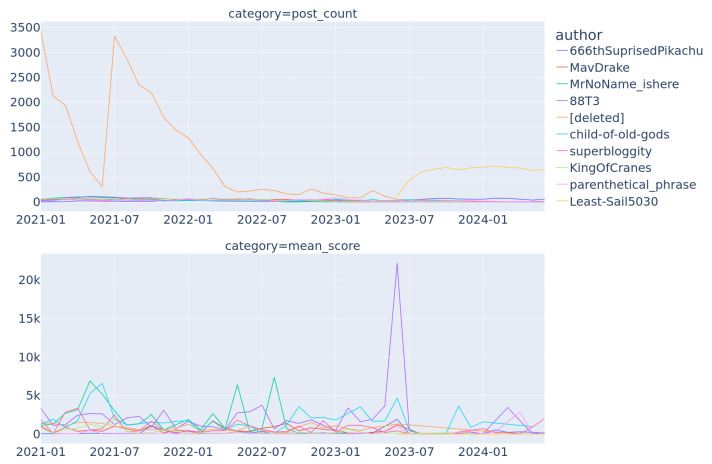
Post count and mean score of top 10 authors (users) by mean score in time.

This research tried to answer the question of whether there was a correlation between the meme’s author and popularity. Figure 4 presents the number of posts and their mean score for the top 10 authors by posted meme counts. There were three particular outliers: users “[deleted]”, “88T3”, and “Least-Sail5030”. The first user was simply a tag assigned to any deleted account, so it did not lead to any particular persona. The second, “88T3”, was an actual user, whose account was suspended in 2023 for “threatening violence” (source post: https://www.reddit.com/r/MLBPowerPros/comments/15nj5m1/this_is_u88t3_my_account_has_been_permanently/ (accessed on 1 February 2025)), but the user was supposedly reactivated. As of publishing this paper, the account remained suspended (account link: https://www.reddit.com/user/88T3/ (accessed 1 Febraury 2025)), but a second account remains active (account link: https://www.reddit.com/user/88T3_2/ (accessed 1 Febraury 2025)). The third user “Least-Sail5030” was supposedly a bot account (source post: https://www.reddit.com/r/InternetMysteries/comments/12bo52g/further_analysis_of_uleastsail5030_the_mysterious/ (accessed on 1 February 2025)), which explains how it averaged 609–724 monthly posts. Interestingly, the bot account used crossposts—a Reddit-specific posting mechanism allowing to “copy” the content from one subreddit to another [26]. That account was also suspended at the time of writing this paper (source post: https://www.reddit.com/user/Least-Sail5030/ (accessed on 1 February 2025)). The remaining accounts, although they had higher than average posts counts and mean scores, were not outstanding enough to draw additional conclusions.

### 5.2. The Top Memes

**Figure 5:**
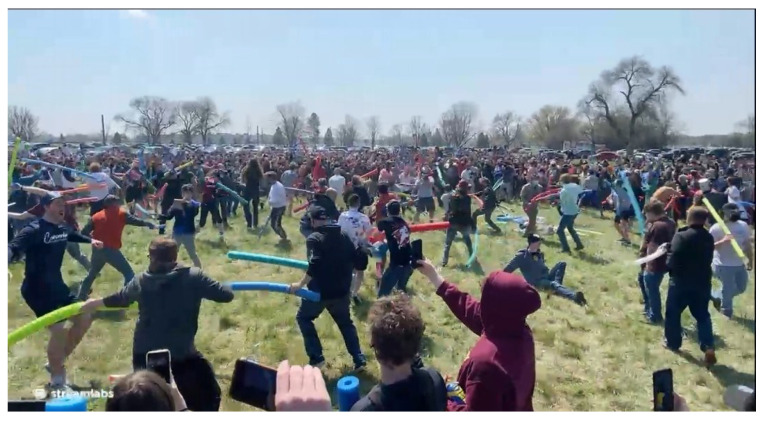
The top post (meme) from r/memes in the time period from January 2021 to July 2024.

Before giving the recipe for creating the best memes, we present the best memes. This was a qualitative approach, where we manually browsed the top posts (by score) within the time range. Figure 5 shows the highest-scored post and meme (https://www.reddit.com/r/memes/comments/mxogvd/they_did_it_they_actually_did_it_battle_of_josh/ (accessed on 1 February 2025)). The post depicts “The battle of Josh”, an event held at Air Park in Lincoln, Nebraska, on 24 April 2021 (source (https://en.wikipedia.org/wiki/Josh_fight (accessed on 1 February 2025))). The post achieved 246,720 upvotes and 4596 comments.

The Josh Fight began as a viral internet meme, evolving into a mock battle and charity fundraiser. The idea originated from Josh Swain, a civil engineering student from Tucson, Arizona, who, out of boredom during the COVID-19 lockdowns on 24 April 2020, created a Facebook Messenger group chat including multiple people named Josh Swain. A screenshot of the chat quickly went viral online. Swain then challenged the participants to meet at a specific location a year later to compete for the right to the name “Josh”. Though initially intended as a joke, the event attracted nearly a thousand attendees. The gathering remained lighthearted, with no real violence involved.

The meme was removed by moderation for breaking rule number 1 of r/memes, i.e., “Rule 1 - ALL POSTS MUST BE MEMES AND NO REACTION MEMES”.

Interestingly, the rest of the top 10 memes (presented in Table 1) on r/memes had no particular similarity when it came to the template, text length, context, or visual style. The manual verifiers were able to derive one distinguishing similarity—the reference to major world or Reddit events. For example, a meme referred to the Ukraine–Russia War (source post: https://www.reddit.com/r/memes/comments/t19inj/ukraine_got_chad_volodymyr_zelensky/ (accessed on 1 February 2025)) (219,059 upvotes), another to the Facebook outage in October 2021 (source post: https://www.reddit.com/r/memes/comments/q1b13o/reddit_might_be_shit_but_its_our_shit/ (accessed on 1 February 2025)) (208,020 upvotes), Reddit and GameStop stocks events [27] (source post 1: https://www.reddit.com/r/memes/comments/l6qbnp/wanna_hear_another_joke/ (accessed on 1 February 2025)) (181,093 upvotes) and source post 2: https://www.reddit.com/r/memes/comments/l7hah3/what_a_shame/ (accessed on 1 February 2025)) (207,859 upvotes) or the beginning of 2021 (source post: https://www.reddit.com/r/memes/comments/ks2asd/its_been_real_fam/ (accessed on 1 February 2025)) (183,612 upvotes). The main conclusion drawn from the analysis of the absolute peak of top subreddits is that timing is extremely important.

Note that the meme “Wait I didn’t mean it like that” was posted twice (two separate posts). We speculated that it was due to a bug (related Reddit thread: https://www.reddit.com/r/memes/comments/lm7wbx/comment/gnwj7lu/?utm_source=share&utm_medium=web3x&utm_name=web3xcss&utm_term=1&utm_content=share_button (accessed on 1 February 2025)).

### 5.3. The Best Meme Templates

**Figure 6:**
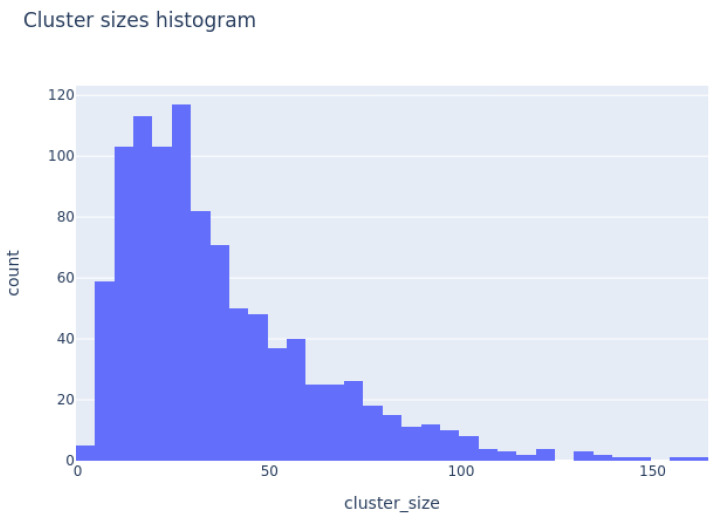
Histogram of the sizes of clusters.

Finally, we arrive at the key finding of this contribution, the best meme templates extracted from the 1000 clusters. K-means clustering produced clusters with a skewed distribution of sizes presented in Figure 6. We noted that most of the clusters were of size between 1 and 100.

The templates were described using the keyword extracted from image captioning, as mentioned in Section 4. Table 2 presents the statistics of these templates.

The top meme templates originated from different works of pop culture (often documented on the KnowYourMeme website (https://knowyourmeme.com/ (accessed on 1 February 2025))). The origins of all memes in the top 10 were:

1. “sign, shirt, board, holding, tie”—originally the character of Jim from a TV series *“The Office”*;
2. “cartoon, woman, picture, hat, pink”—characters from a TV series *“The Family Guy”*;
3. “bus, school, pictures, train, tracks”—screenshots from a YouTube Video (https://www.youtube.com/watch?v=poomuKzSGZA—accessed on 1 February 2025);
4. “suit, tie, picture, man, close”—Mince McMahon during an episode of World Wrestling Entertainment;
5. “cartoon, tuxedo, pooh, winnie, caption”—a modified version of the character Winnie the Pooh from *“Winnie the Pooh and Tigger Too”*;
6. “cartoon, woman, man, beard, expressions”—a variation of a “Wojak Comic”;
7. “cartoon, tuxedo, bear, picture, man”—a modified version of the character Winnie the Pooh from *“Winnie the Pooh and Tigger Too”*;
8. “cartoon, screen, standing, person, quote”—the character Lisa Simpson from a TV series *“The Simpsons”*;
9. “cartoon, path, picture, castle, sign”—a reference to a card from a TV series and card game *“Yu-Gi-Oh!”*;
10. “monkey, cartoon, shirt, green, caption”—a character of a Monkey Puppet named Kenta from a TV series *“Ōkiku Naru Ko”*.

The most prevalent origin among the top meme templates appeared to be various television series, encompassing both ongoing productions and shows that had concluded.

Turning to the numerical statistics, notable discrepancies emerged in the mean scores. The highest mean score surpassed 20,000 upvotes, whereas the lower end of the ranking averaged around half of that, with just over 11,000 upvotes per meme. There was no clear correlation between cluster size and mean score, as all clusters contained between 52 and 88 images. These cluster sizes might be insufficient to comprehensively represent each category, posing the most significant limitation of this analysis. Achieving an optimal balance between cluster size and the mean cosine similarity (used to model in-cluster consistency) presented a challenge. In this case, greater emphasis was placed on cluster consistency, prioritizing higher in-cluster cosine similarity over larger cluster sizes. To mitigate this limitation, subsequent steps involved identifying overarching trends and patterns across clusters to synthesize the findings.

Within these clusters, distinct groups could be identified based on their format. The first group was idea “presentation” (memes 1, 8), where creators conveyed their perspective, whether it was a controversial viewpoint or a simple expression of an idea or opinion. The second group was “comparison” (memes 2, 6, 9), characterized by memes that juxtaposed two contrasting elements to highlight their differences. The third group was “scaling” (memes 4, 5, 7), where authors illustrated a phenomenon by progressively increasing its magnitude, typically starting from a minor instance and culminating in an extreme example.

A noteworthy observation was the occurrence of the “Winnie-the-Pooh” template in two separate clusters, each containing distinct memes based on the same template. On one hand, this underscored the limitations of the detection method, as the combined use of vector embeddings and clustering did not always yield flawless results, particularly when applied to complex datasets such as Reddit [26]. On the other hand, this phenomenon prompted a deeper inquiry into the nature of meme templates. Although a general definition was provided at the beginning of this paper, this finding invites further exploration of the underlying concept.

### 5.4. What Is a “Meme Template”?—Discussion

Let us delve into a discussion that sets the stage for further research on memes. While the definition of memes has been explored in the existing literature [7,28,29,30], the concept of “meme templates” remains somewhat ambiguous. We aim to clarify this distinction through examples.

**Figure 7:**
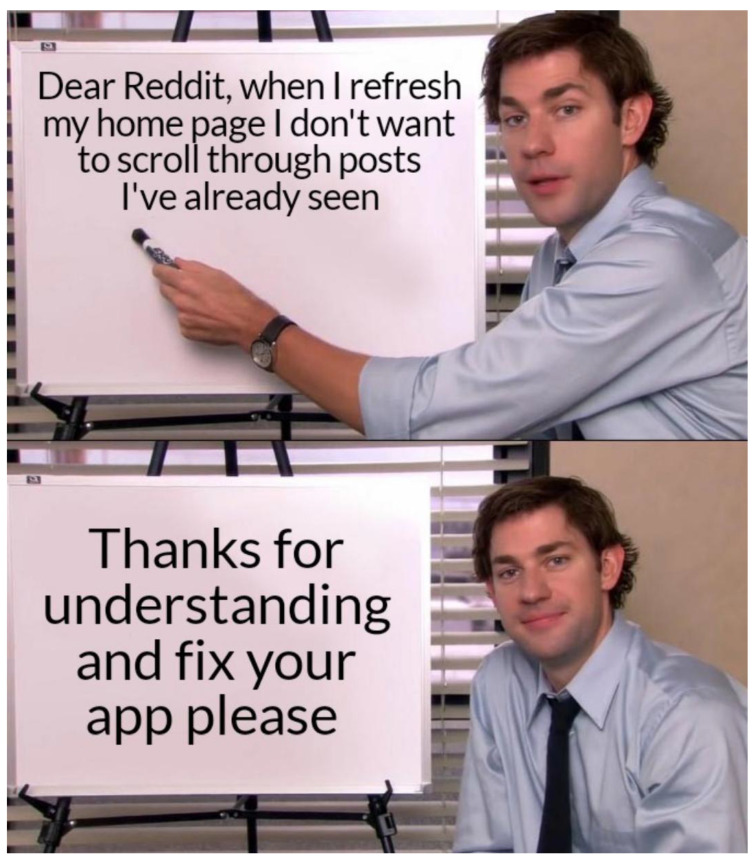
An example meme from the template with keywords: sign, shirt, board, holding, tie (https://www.reddit.com/r/memes/comments/tn4k1q/do_you_remember_any_update_that_didnt_fuck_up_the (accessed on 1 February 2025)).

**Figure 8:**
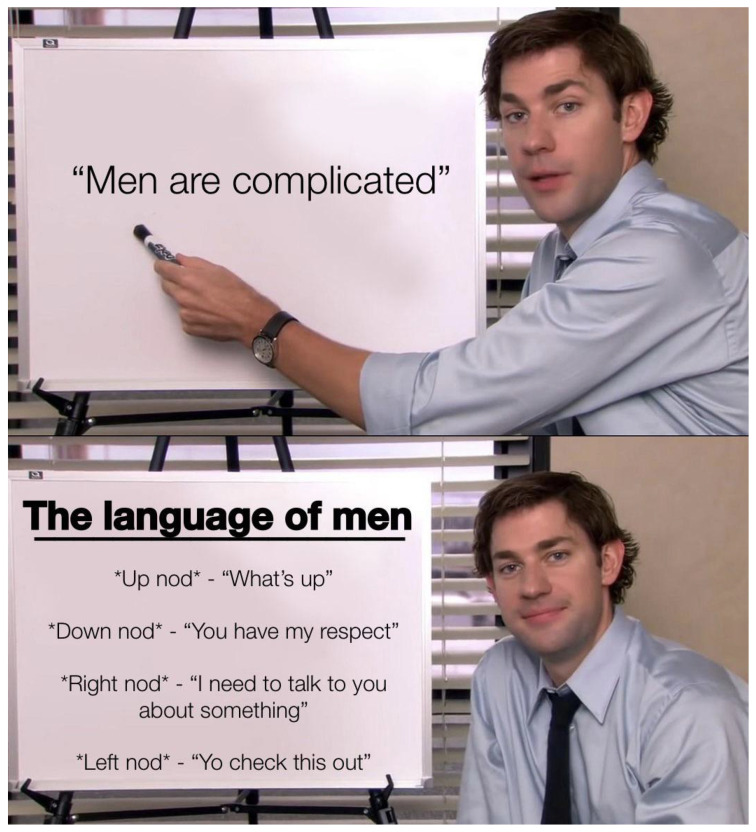
An example meme from the template with keywords: sign, shirt, board, holding, tie (https://www.reddit.com/r/memes/comments/uhhhey/the_secret_language_of_men/ (accessed on 1 February 2025)).

First, meme templates may exhibit minor formatting variations, such as changes in resolution, font style, text length, or color. For instance, see Figure 7 and Figure 8. These differences are subtle and do not significantly impact the difficulty of meme classification.

**Figure 9:**
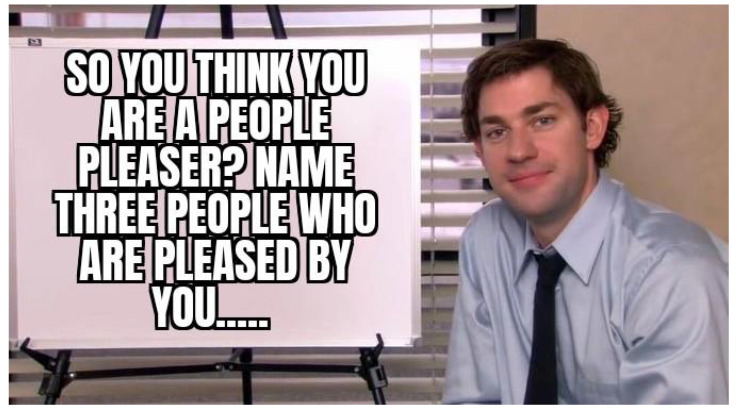
An example meme from the template with keywords: sign, shirt, board, holding, tie (https://www.reddit.com/r/memes/comments/1aujou2/nobody_is_pleased_by_you_live_your_life_without/ (accessed on 1 February 2025)).

A more substantial variation involves the “extension or reduction” of the original template format. For example, Figure 9 can be considered a reduced version of the template shown in Figure 7. The decision to extend or reduce a template often depends on the popularity of the format; in this case, Figure 7 represents a meme derived from the more popular template.

**Figure 10:**
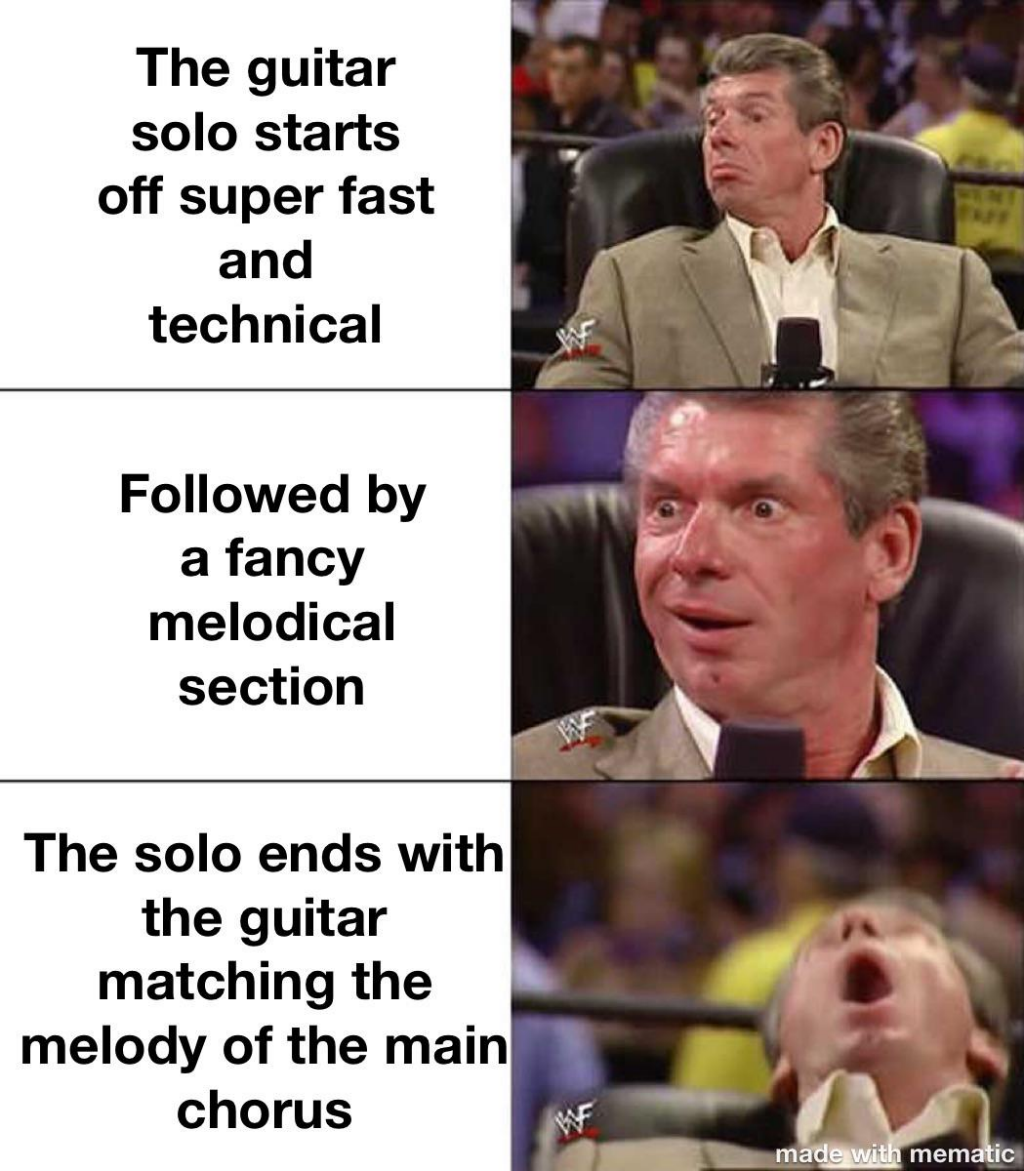
An example meme from the template with keywords: suit, tie, picture, man, close (https://www.reddit.com/r/memes/comments/1adcfez/what_are_some_of_the_best_examples_of_this/ (accessed on 1 February 2025)).

**Figure 11:**
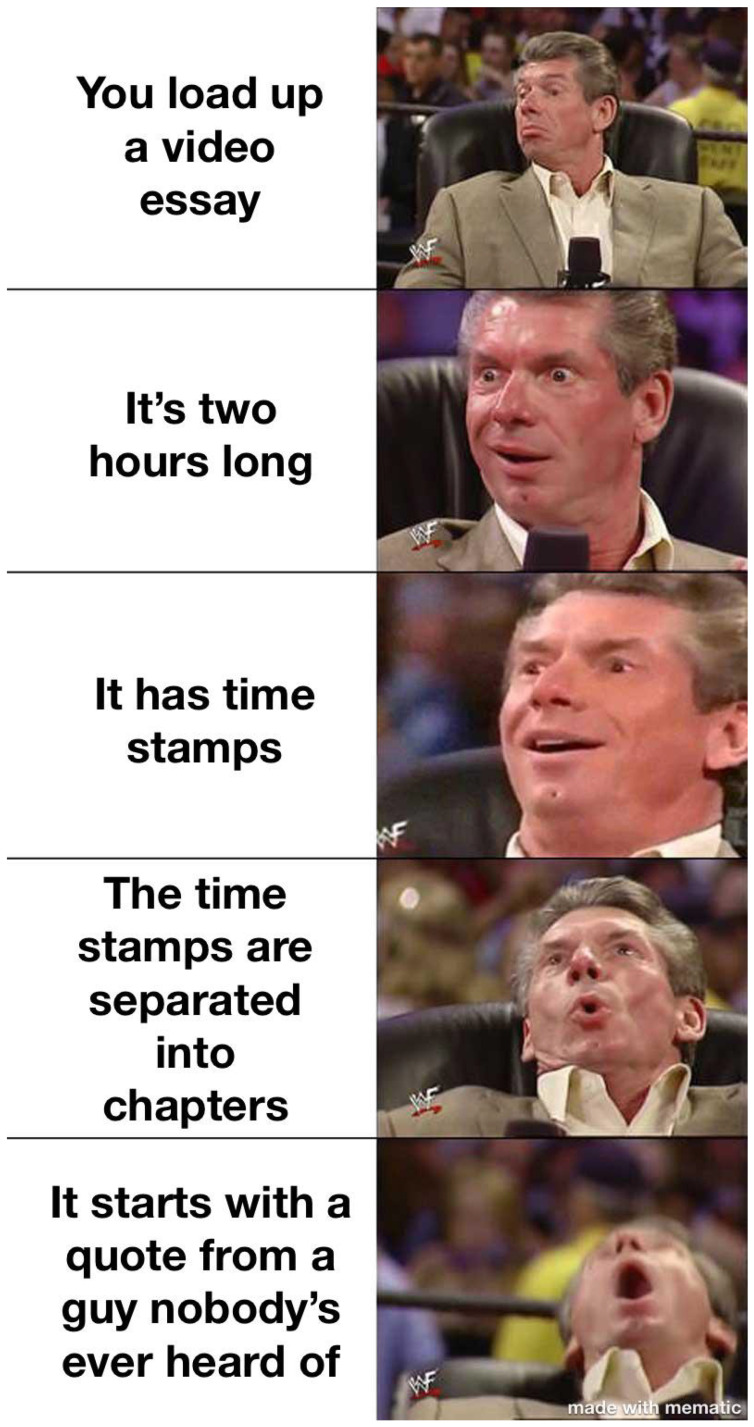
An example meme from the template with keywords: suit, tie, picture, man, close (https://www.reddit.com/r/memes/comments/194pviu/just_what_you_want_on_a_friday_night/ (accessed on 1 February 2025)).

**Figure 12:**
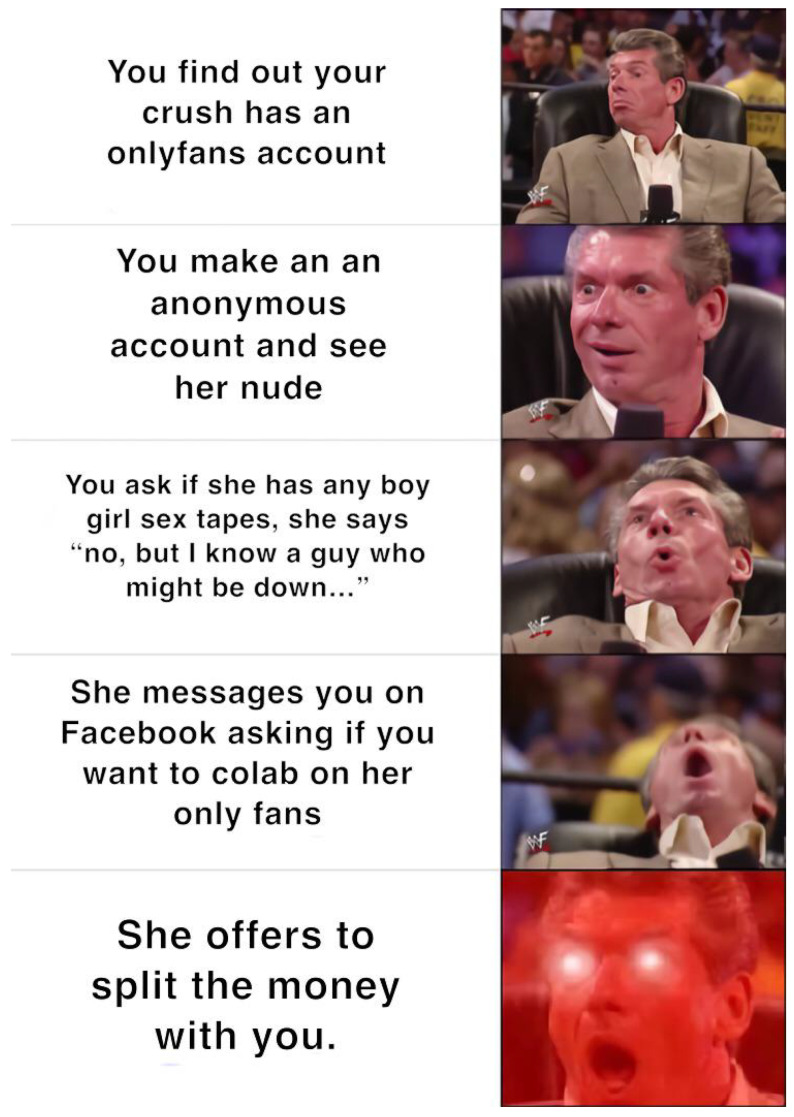
An example meme from the template with keywords: suit, tie, picture, man, close (https://www.reddit.com/r/memes/comments/1cevh7z/ive_done_the_first_4_but_never_with_the_same_chick/ (accessed on 1 February 2025)).

The extension can be used as simplification (cutting a panel from the meme) but also scaling of the original meme. For example, Figure 10, Figure 11 and Figure 12 shows how the memes can be scaled. Importantly, the scaling does not have to follow the same panels (see panels in memes in Figure 10 and Figure 11.

**Figure 13:**
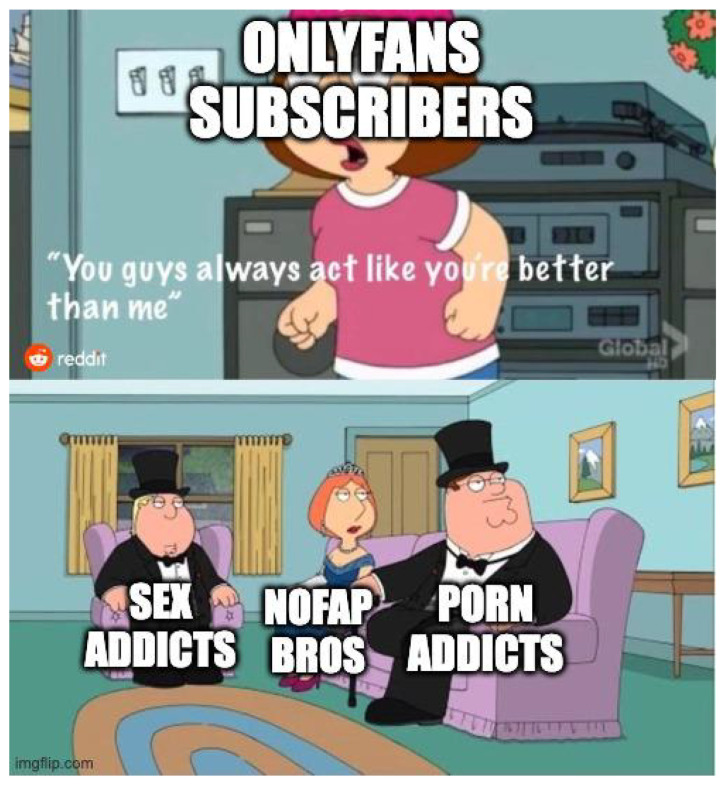
An example meme from the template with keywords: cartoon, woman, picture, hat, pink (https://www.reddit.com/r/memes/comments/1b7etqx/who_tf_pays_for_that/ (accessed on 1 February 2025)).

**Figure 14:**
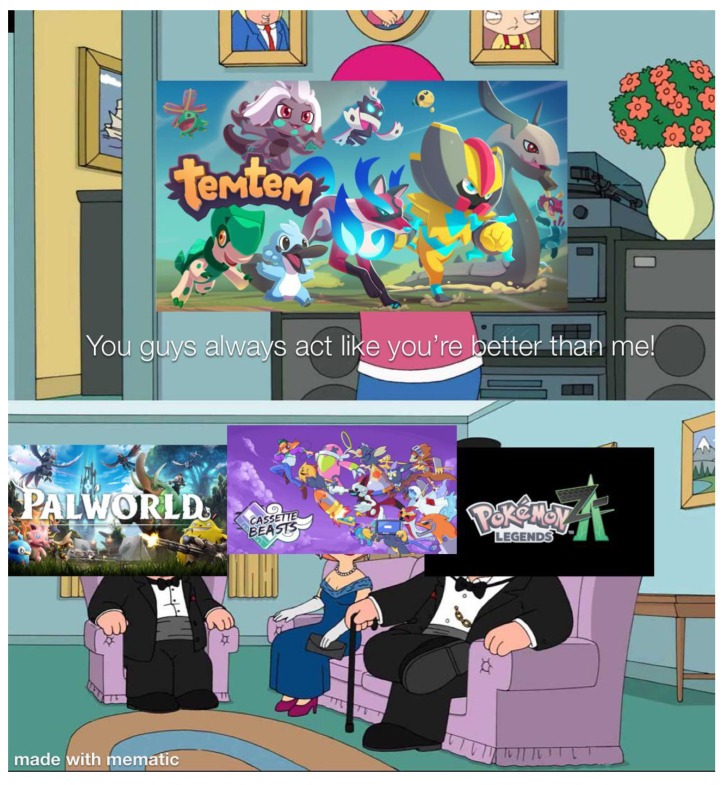
An example meme from the template with keywords: cartoon, woman, picture, hat, pink (https://www.reddit.com/r/memes/comments/1bng0dq/well_the_ceo_of_temtem_like_to_insult_its/ (accessed on 1 February 2025)).

The second type of meme template variation involves modifying the image itself, not just the text. For instance, the template shown in Figure 13 represents a more popular variation. In contrast, Figure 14 illustrates a less popular but more specific visual edit, where the author conveys meaning through image elements rather than using captions.

**Figure 15:**
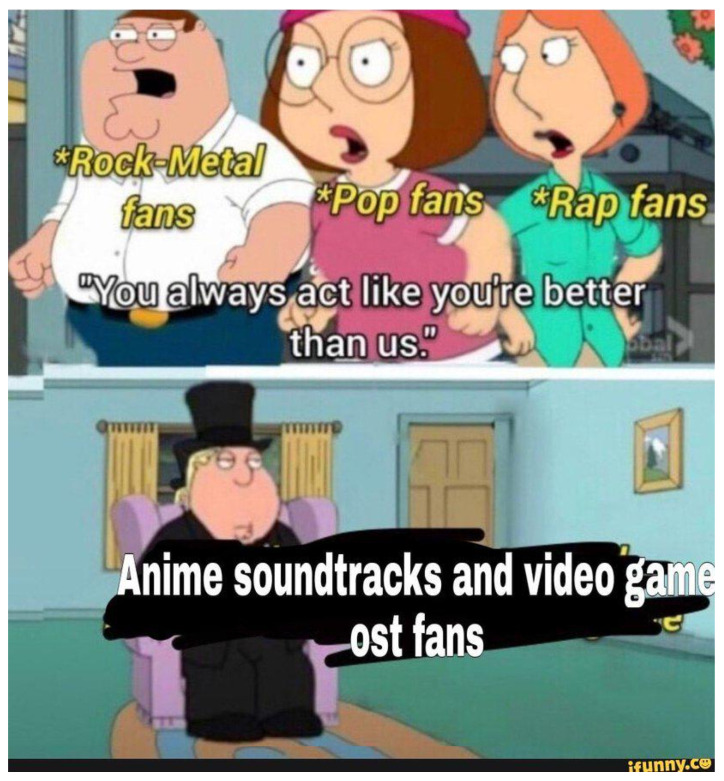
An example meme from the template with keywords: cartoon, woman, picture, hat, pink (https://www.reddit.com/r/memes/comments/18x856g/lost_woods_better_than_any_crap_song_change_my/ (accessed on 1 February 2025)).

Another interesting variation is related to a reversal or inversion of the standard template. See, for example, Figure 15, which reverses the template seen in Figure 13.

Considering the origins and similarities between meme templates, one can observe that despite the variety in templates, the ideas they convey are typically straightforward and often reference elements of pop culture.

### 5.5. Limitations

Despite the promising findings, our study has several limitations. First, the dataset used included a significant gap, as discussed in Section 3, which may have affected the generalizability of our results.

Additionally, while we employed state-of-the-art models, namely, ViT and BLIP, these models had been established in the literature for years and may have carried inherent biases. They were not specifically designed for meme analysis, which could impact their performance. We deliberately chose not to compare multiple models, as our goal was to focus on tangible results and ensure interpretability of top outcomes rather than conduct a broad model evaluation.

Another key limitation is the lack of a golden standard for evaluation, making it difficult to validate our results definitively. Instead, we relied on the trustworthiness of our selected models, methods, and dataset quality. Furthermore, the K-means clustering algorithm can introduce potential bias depending on the chosen number of clusters. While we determined that number based on the Elbow method and in-cluster cosine similarity of image embeddings, alternative choices could yield different insights.

Finally, while we manually described template similarity, a more comprehensive validation through consultation with a larger pool of experts and users would enhance the robustness of our findings.

Despite these limitations, our study prioritized delivering tangible and interpretable results, providing valuable insights into meme analysis using deep learning techniques.

## 6. Conclusions

Our findings suggest that achieving top-tier meme popularity (top 1%) is not strongly correlated with the choice of template. There was a substantial gap between the most popular memes (reaching over 100,000 upvotes) and the mean scores of the most popular templates (10,000–20,000 upvotes). To achieve peak internet attention, a meme must be well timed and reference significant historical events.

To consistently achieve high scores, users should consider two key aspects. The first is the choice of meme template. The average scores of these templates are notably high, considering that most posts on Reddit (in some subreddits, over 90%) receive little to no attention [26]. The second is the conveyed idea. The most successful templates often employ simple idea presentation, comparisons, or emotional scaling.

Overall, our findings suggest that while template selection is not the sole determinant of virality, it plays a significant role in achieving moderate success. However, this analysis has certain limitations that we intend to address in future research.

A primary area for further investigation is textual analysis, which can be conducted using established text extraction methods. Additionally, analyzing temporal trends could provide a more comprehensive understanding of meme dynamics. Examining low-engagement (“bottom”) memes may also offer valuable insights by serving as a counterpoint to highly viral content. While user attention can be inferred relatively easily through engagement metrics such as scores or comments, understanding why certain memes fail to engage users presents a greater challenge.

Finally, a key limitation of our study was the reliance on a single image embedding model. Future research could explore multiple embedding models to determine whether model-specific interactions influence results. Comparing these approaches would enhance our understanding of the robustness and generalizability of different image embedding techniques.

Beyond these methodological considerations, future work should also investigate meme virality in real-world meme generation scenarios. One concrete direction is to test meme templates in controlled experiments, where variations in text, imagery, and format are systematically altered to measure their impact on user engagement. Conducting such experiments on social media platforms or within simulated environments would provide valuable empirical insights into the factors driving meme success. By bridging computational analysis with real-world testing, future studies can offer a more holistic understanding of meme propagation dynamics.

## Figures and Tables

**Table 1:** Top 10 memes on Reddit considered during the time period and their relevant world events.

Title	Score	Comments	Related Event
They DID It ! They actually did it ! Battle of Josh (https://reddit.com/r/memes/comments/mxogvd/they_did_it_they_actually_did_it_battle_of_josh/ (accessed on 1 February 2025))	246,720	4596	“The Battle of Josh”
Ukraine got CHAD Volodymyr Zelensky.[emojis] (https://reddit.com/r/memes/comments/t19inj/ukraine_got_chad_volodymyr_zelensky/ (accessed on 1 February 2025))	219,059	2727	Russo-Ukrainian War (https://en.wikipedia.org/wiki/Russo-Ukrainian_War (accessed on 1 February 2025))
Wait I didn’t mean it like that (https://reddit.com/r/memes/comments/lkodmq/wait_i_didnt_mean_it_like_that/ (accessed on 1 February 2025))	208,512	1531	-
Reddit might be shit but it’s our shit. (https://reddit.com/r/memes/comments/q1b13o/reddit_might_be_shit_but_its_our_shit/ (accessed on 1 February 2025))	208,020	1518	2021 Facebook outage (https://en.wikipedia.org/wiki/2021_Facebook_outage (accessed on 1 February 2025))
What a shame (https://reddit.com/r/memes/comments/l7hah3/what_a_shame/ (accessed on 1 February 2025))	207,859	1137	GameStop short squeeze (https://en.wikipedia.org/wiki/GameStop_short_squeeze (accessed on 1 February 2025))
Wait I didn’t mean it like that (https://reddit.com/r/memes/comments/lm7wbx/wait_i_didnt_mean_it_like_that/ (accessed on 1 February 2025))	198,588	1547	-
Team monke (https://reddit.com/r/memes/comments/m2y08v/team_monke/ (accessed on 1 February 2025))	195,971	1570	-
These are confusing times (https://reddit.com/r/memes/comments/kxrxuv/these_are_confusing_times/ (accessed on 1 February 2025))	191,965	1140	-
It’s been real fam. (https://reddit.com/r/memes/comments/ks2asd/its_been_real_fam/ (accessed on 1 February 2025))	183,612	2049	COVID-19 pandemic (https://pl.wikipedia.org/wiki/COVID-19 (accessed on 1 February 2025))
Wanna hear another joke (https://reddit.com/r/memes/comments/l6qbnp/wanna_hear_another_joke/ (accessed on 1 February 2025))	181,093	1859	GameStop short squeeze (https://en.wikipedia.org/wiki/GameStop_short_squeeze (accessed on 1 February 2025))

**Table 2:** Statistics and example memes from the top 10 meme templates.

Keywords	Cluster Size	Means Score in Cluster	Mean Cosine Similarity	Title
Sign, shirt, board, holding, tie	73	20,743	0.85	Just enjoy the game (https://reddit.com/r/memes/comments/1avtxiw/just_enjoy_the_game/ (accessed on 1 February 2025))
Cartoon, woman, picture, hat, pink	52	17,192	0.72	How wars start has always seemed stupid to me (https://reddit.com/r/memes/comments/1dmdz9p/how_wars_start_has_always_seemed_stupid_to_me/ (accessed on 1 February 2025))
Bus, school, pictures, train, tracks	59	15,409	0.80	The pain of introverts (https://reddit.com/r/memes/comments/1cfwsed/the_pain_of_introverts/ (accessed on 1 February 2025))
Suit, tie, picture, man, close	58	15,187	0.74	My favorite architect (https://reddit.com/r/memes/comments/1d40nrn/my_favorite_architect/ (accessed on 1 February 2025))
Cartoon, tuxedo, pooh, winnie, caption	71	15,089	0.79	Not to mention women who are into dad bods (https://reddit.com/r/memes/comments/1dbvjs2/not_to_mention_women_who_are_into_dad_bods/ (accessed on 1 February 2025))
Cartoon, woman, man, beard, expressions	88	13,869	0.73	Woman and Chad (https://reddit.com/r/memes/comments/1cut6gw/woman_and_chad/ (accessed on 1 February 2025))
Cartoon, tuxedo, bear, picture, man	67	12,863	0.85	Optimize (https://reddit.com/r/memes/comments/1bujeu6/optimize/ (accessed on 1 February 2025))
Cartoon, screen, standing, person, quote	59	11,552	0.85	Somebody had to say it (https://reddit.com/r/memes/comments/1cbs25o/somebody_had_to_say_it/ (accessed on 1 February 2025))
Cartoon, path, picture, castle, sign	69	11,320	0.86	Which one is it? (https://reddit.com/r/memes/comments/1bo9rnp/which_one_is_it/ (accessed on 1 February 2025))
Monkey, cartoon, shirt, green, caption	56	11,312	0.73	Apparently you weren’t supposed to do that (https://reddit.com/r/memes/comments/194ootv/apparently_you_werent_supposed_to_do_that/ (accessed on 1 February 2025))

## Data Availability

Data are contained within the article.
